# The Role of Femoroacetabular Impingement in Core Muscle Injury/Athletic Pubalgia: Diagnosis and Management

**DOI:** 10.3389/fsurg.2016.00006

**Published:** 2016-02-12

**Authors:** David S. Strosberg, Thomas J. Ellis, David B. Renton

**Affiliations:** ^1^Department of Surgery, The Ohio State University Wexner Medical Center, Columbus, OH, USA; ^2^Orthopedic One, Columbus, OH, USA; ^3^Center for Minimally Invasive Surgery, The Ohio State University Wexner Medical Center, Columbus, OH, USA

**Keywords:** core muscle injury, athletic pubalgia, femoroacetabular impingement, sports hernia

## Abstract

Chronic groin pain in athletes represents a major diagnostic and therapeutic challenge in sports medicine. Two recognized causes of inguinal pain in the young adult athlete are core muscle injury/athletic pubalgia (CMI/AP) and femoroacetabular impingement (FAI). CMI/AP and FAI were previously considered to be two distinct entities; however, recent studies have suggested both entities to frequently coincide in the athlete with groin pain. This article briefly discusses the role of FAI in CMI/AP and the diagnosis and management of this complex disease.

## Introduction

Chronic groin pain in athletes represents a major diagnostic and therapeutic challenge in sports medicine. Athletic injuries to the hip and groin occur less frequently than injuries to the lower extremities (1); however, the complexity of the anatomy and biomechanics of the groin makes these injuries more difficult to identify and manage. Patients may find themselves evaluated by multiple physicians and receive numerous diagnostic studies over a period of months. Early and accurate diagnosis is essential for return to play and improved quality of life (2).

Two recognized causes of an insidious onset of groin pain in the young adult athlete are core muscle injury/athletic pubalgia (CMI/AP) and femoroacetabular impingement (FAI). CMI/AP, or “sports hernia,” is a syndrome of disabling exertional inguinal and adductor pain commonly seen in high-performance athletes, possibly due to a disruption of the musculature of the posterior inguinal wall (2, 3). FAI or hip impingement syndrome may also cause inguinal pain in the athlete, whereby abnormal contact between the femoral head and acetabular junction results in chondral and labral injury (4).

While CMI/AP and FAI were previously considered to be two distinct entities, recent studies have suggested both entities to frequently coincide in the athlete with groin pain. Our primary objectives are to discuss the role of FAI in CMI/AP, and to discuss the diagnosis and management of this complex disease.

## Core Muscle Injury/Athletic Pubalgia and Femoroacetabular Impingement

**Figure 1:**
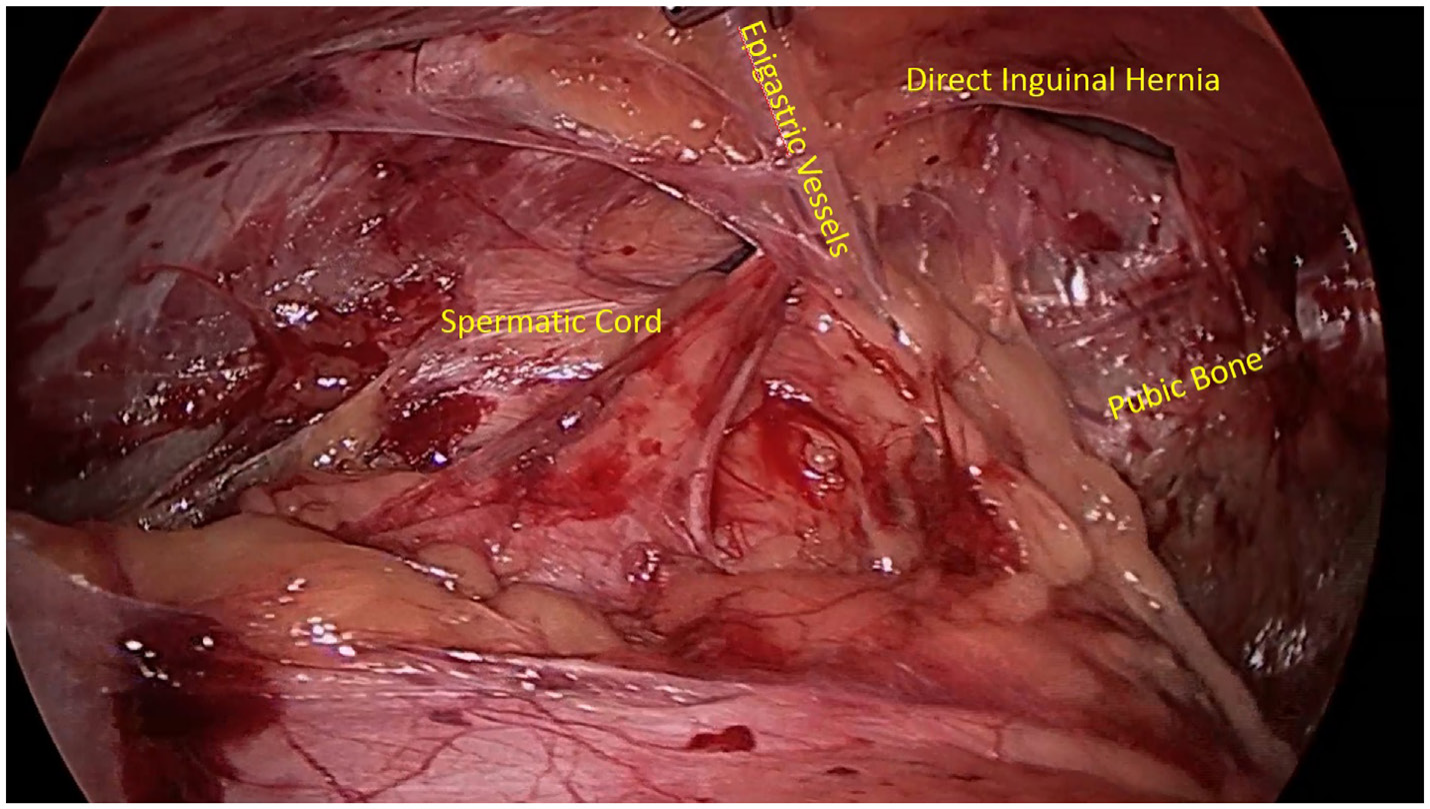
**Laparoscopic view of the inguinal abdominal wall musculature with the presence of a direct inguinal hernia**.

A comprehensive understanding of normal inguinal anatomy is critical to the diagnosis and management of groin pain. The anterior abdominal wall consists of the external oblique muscle and aponeurosis, internal oblique muscle and aponeurosis, transversus abdominis muscle and aponeurosis, and transversalis fascia. Medially, the rectus abdominis muscle fibers insert on the pubic tubercle. The inguinal canal extends between the deep inguinal and superficial inguinal rings, or defects in the aforementioned aponeuroses and fascia, and contains the spermatic cord in men and round ligament in women. Clinical hernias develop with a protrusion of omentum, fat, or bowel through the inguinal canal (indirect hernias), through the floor of a weakened abdominal wall musculature medial to the epigastric vessels (direct hernias), or below the inguinal ligament (femoral hernias). There are multiple notable nerves in the inguinal region, including the iliohypogastric nerve, the ilioinguinal nerve, and the genital branch of the genitofemoral nerve. The iliohypogastric nerve travels on the anterior surface of the internal oblique muscle and aponeurosis just medial and cranial to the deep ring. The ilioinguinal nerve runs anterior to the spermatic cord in the inguinal canal, and the genital branch of the genitofemoral nerve travels through the inguinal canal with the spermatic cord. A laparoscopic view of the inguinal anterior abdominal wall is shown with the presence of a direct inguinal hernia passing medially to the epigastric vessels (Figure 1).

Core muscle injury/athletic pubalgia is also known as a sports or sportsman’s hernia, however, the term “hernia” may be a misnomer as there is no true protrusion through a defect in the anterior abdominal wall. There is no consensus for the etiology of CMI/AP, although most theories support an overuse syndrome. Repetitive pelvic motion against a fixed extremity may produce a shearing force across the pubic symphysis, leading to attenuation, avulsion, or tearing of the musculature or fascia of the posterior inguinal wall (1). It has been suggested that CMI/AP may result in disruption of the origin of the rectus abdominis muscle from the pubic tubercle, avulsion of the internal oblique muscle fibers at the pubic tubercle, or an abnormality in the external oblique aponeurosis (2, 3). Another theory postulates a laxity in the posterior inguinal wall that stretches under force, entrapping the nerves along the inguinal floor, including the genital branch of the genitofemoral nerve, ilioinguinal, lateral femoral cutaneous, or obturator nerves (5).

**Figure 2:**
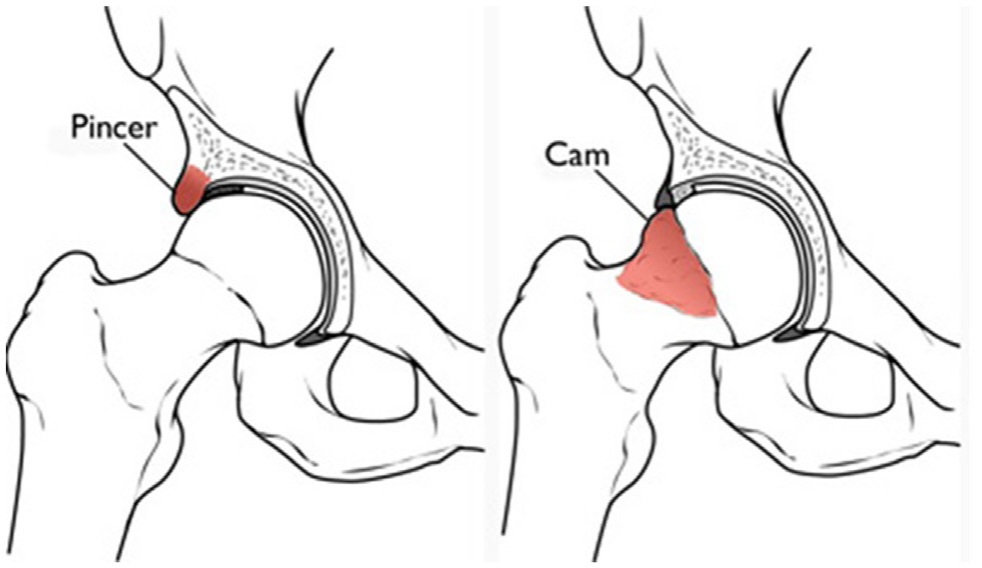
**Pincer and cam impingement in femoroacetabular impingement (Reproduced with permission from the American Academy of Orthopedic Surgeons)**.

Femoroacetabular impingement represents abnormal contact between the femoral head and acetabular junction in the young active adult population, ultimately causing patterns of chondral and labral injury. There are two main variations: pincer and cam lesions (Figure 2). Pincer impingement involves over coverage of the femoral head by the acetabulum due to focal rim lesions or cephalad retroversion (4, 6). The labrum is frequently injured with the abnormal contact of the femoral neck and acetabular rim. A cam lesion is an osteochondral prominence at the femoral head–neck junction leading to loss of the normal femoral head–neck offset. Cam lesions are most commonly anterolateral, and affect the anterosuperior chondrolabral junction. This results in chondrolabral separation and frequently delamination of the adjacent acetabular chondral surface (4, 6). The alpha angle, or the angle between the femoral neck axis and a line through a point where the contour of the femoral head–neck junction exceeds the radius of the femoral head, is considered diagnostic of cam impingement when greater than 50–55° (7).

The relationship between CMI/AP and FAI has been a recent area of interest. Loss of clearance between the femoral neck and acetabular rim with FAI may restrict terminal motion of the hip in multiple planes. This restriction in the high-performance athlete may lead to compensatory stresses on the lumbar spine, pubic symphysis, sacroiliac joint, and posterior acetabulum (3, 4). Excessive biomechanical stress on the groin may lead to secondary injury to the abdominal wall musculature, including the posterior inguinal wall, resulting in symptomatic CMI/AP; however, the biomechanical relationship has yet to be elucidated in this theorem. The muscles typically affected by FAI include the adductor longus, proximal hamstrings, abductors, iliopsoas, and hip flexors (4).

Recent studies have demonstrated an association between CMI/AP and FAI. Hammoud et al. described a consecutive series of 38 professional athletes who had been treated for symptomatic FAI, with 12 (32%) of those patients identified as having previous surgical intervention for CMI/AP. After additional treatment for FAI, all 12 patients were subsequently able to return to play. Additionally, of the 26 remaining patients, 15 had symptoms of CMI/AP that resolved with isolated treatment of their hip pathology. In this study, 39% (15/39) of athletes with both CMI/AP and FAI symptoms had complete resolution of pain and return to field with FAI surgery alone (4).

Another retrospective review by Economopoulos et al. investigated radiographic evidence of FAI in patients who had been treated for CMI/AP. Athletes who underwent surgical intervention for CMI/AP were found to have concomitant radiographic evidence of FAI in at least 1 hip in 37 of 43 patients (86%) (6). Cam lesions were identified by measurement of the alpha angle on frog-leg lateral radiographs, with an alpha angle of 55° or greater considered positive for FAI. Cam lesions were identified in 84% and pincer lesions in 28% of hip radiographs (6).

## Diagnosis

The diagnosis of CMI/AP is difficult to make given its insidious onset and variable physical exam findings (8). Many patients cannot recall when the specific injury occurred, and nearly all patients will completely stop their competitive level of activity by the time of evaluation (9). Pain is typically located in the inguinal canal near the insertion of the rectus abdominis on the pubis, and worsens with activity and alleviated with rest. Pain will often extend down the internal thigh/lateral portion of the scrotum/perineum during activity. Sneezing, coughing, or Valsalva maneuvers may worsen the pain in up to 10% of patients, despite the lack of a palpable bulge on examination (2, 9). Physical examination may also reveal pubic tubercle tenderness, with point tenderness over the medial aspect of the inguinal ligament before its insertion into the pubic tubercle (10). The examiner will elicit tenderness in 88% of patients with resisted adduction of the hip, and pain with a resisted sit-up is common (1, 9).

The examiner should also evaluate the hip joint for clinical signs and symptoms of FAI in this patient population. Patients with FAI will exhibit hip and groin pain with flexion and internal rotation of the hip, and some patients may experience limited hip internal rotation, flexion, and abduction (8, 10). The anterior impingement test, or pain with hip flexion, adduction, and internal rotation, may also suggest hip pathology (8).

If suspecting underlying FAI in a patient with CMI/AP, plain radiographs of the anteroposterior pelvis of both hips and lateral view of the proximal femur should be performed to evaluate pathology and the femoral head–neck junction and acetabulum. Magnetic resonance imaging (MRI) is the only imaging modality useful in the diagnosis of CMI/AP, as radiographs are often negative (10). Disruption or cleft sign at the rectus abdominis or adductor aponeurosis at the anterior pelvis is suggestive of CMI/AP (8). In the literature, 9 to 65% of MRIs will identify a tear of the rectus abdominis muscle at the insertion on the pubis in patients with symptoms of CMI/AP. However, there is a higher incidence of non-specific radiographic signs, including small avulsion fractures, unexplained edema, and musculotendinous asymmetry (9, 11). Compared with findings at surgery, MRI is 68% sensitive and 100% specific for rectus abdominis disruptions, and 86% sensitive and 89% specific for adductor pathology (11).

The authors routinely use plain radiographs and dedicated hip CTs to determine amount of bony deformity and whether the FAI is due to an isolated cam deformity, isolated pincer deformity, or components of both. They also look at the femoral version and the prominence of the anterior inferior iliac spine (AIIS), as the presence of those in combination with pain with straight hip flexion is suggestive of impingement of the anterior facet of the trochanter on the AIIS, known as extra-articular impingement. For patients who have failed core and gluteal strengthening programs, MRI is useful to evaluate the hip and pubic symphysis as an adjunct diagnostic tool.

## Management

As with many disease processes, non-surgical options should be considered prior to surgical intervention of CMI/AP or FAI. Non-narcotic analgesics during game play can be considered if the athlete is willing to participate with high levels of pain for the short term. Intra-articular corticosteroid injections may be considered in the professional athlete with CMI/AP to help complete a season; however, there is little literature on its efficacy (8). Platelet-rich plasma (PRP) intra-articular injection has been shown to decrease immediate postoperative pain following arthroscopic hip surgery for FAI in a randomized control trial; however, the long-term advantage is uncertain (12). The senior author of this paper has abandoned the use of corticosteroids for CMI/AP due to the lack of lasting relief, and routinely injects PRP for chronic adductor pain.

Physical therapy has shown a 27% success rate for return to sports activities after 3 months of therapy in CMI/AP (13), and should be attempted if symptoms began <2 months at presentation. Physical therapy should be aimed at strengthening of the muscles that stabilize the pelvis, hip joints, lower abdominal musculature, and adductor muscles. Most athletes who have failed conservative management will elect surgical intervention. Surgical intervention should be considered if physical therapy fails after 2 months in the professional athlete, and 4–6 months in non-professional athlete patients (13).

Conservative treatment for FAI should be aimed at core strengthening, non-steroidal anti-inflammatory medications (NSAIDs), and physical therapy. Therapy should optimize the alignment and range of motion of the hip joint, while avoiding passive stretches that may exacerbate symptoms (7).

**Figure 3:**
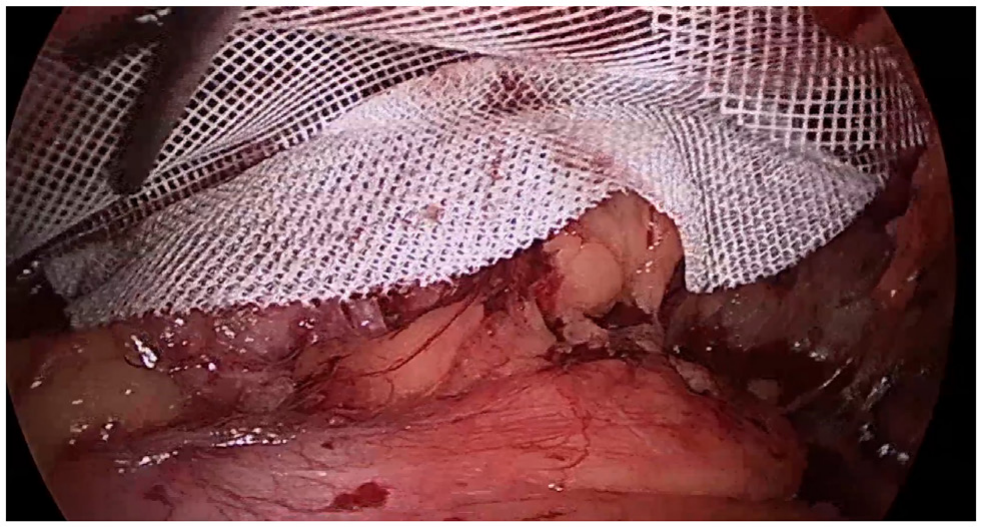
**Laparoscopic view of the inguinal abdominal wall musculature following mesh placement**.

Nearly all studies have demonstrated surgical correction for CMI/AP to be more effective for relief of pain and earlier return to field (5, 9, 13–15). Described techniques for the general surgeon include open tissue repair, tension-free repair with mesh, and laparoscopic total extra pre-peritoneal inguinal hernia repair (TEP) (2). Open inguinal hernia repair for CMI/AP aims to reinforce areas of laxity along the inguinal floor and to restore the rectus abdominis muscle to its origin at the pubic tubercle, rather than re-approximate or recreate the internal ring (9). Alternatively, TEP allows for visualization of the posterior inguinal wall musculature and placement of a large mesh to distribute forces over a greater surface area (Figure 3). Implementing hernia repair techniques in CMI/AP has a high success rate, as athletes who underwent open tissue or tension-free repair with mesh returned to sports 80–95% of the time, and those who underwent laparoscopic repair with mesh had a 93–100% return rate (8). Patients with CMI/AP who underwent laparoscopic repair returned to sports at mean 3 weeks, compared to 5 weeks postoperatively for open tissue or mesh repairs (16).

Adductor release is a newer option in the treatment of CMI/AP. Given the opposing force of the adductor on the pelvis, this technique involves cutting the insertion of the adductor longus muscles from its insertion on the pubic bone (9). The tendon is released from its insertion approximately 1 cm from the pubis. A retrospective study by Rossidis et al. investigated 54 patients with CMI/AP symptoms who underwent combined laparoscopic TEP inguinal hernia repair and ipsilateral adductor longus tenotomy. All were able to return to full sports activity in a mean of 24 days (range 21–28 days) (14).

**Figure 4:**
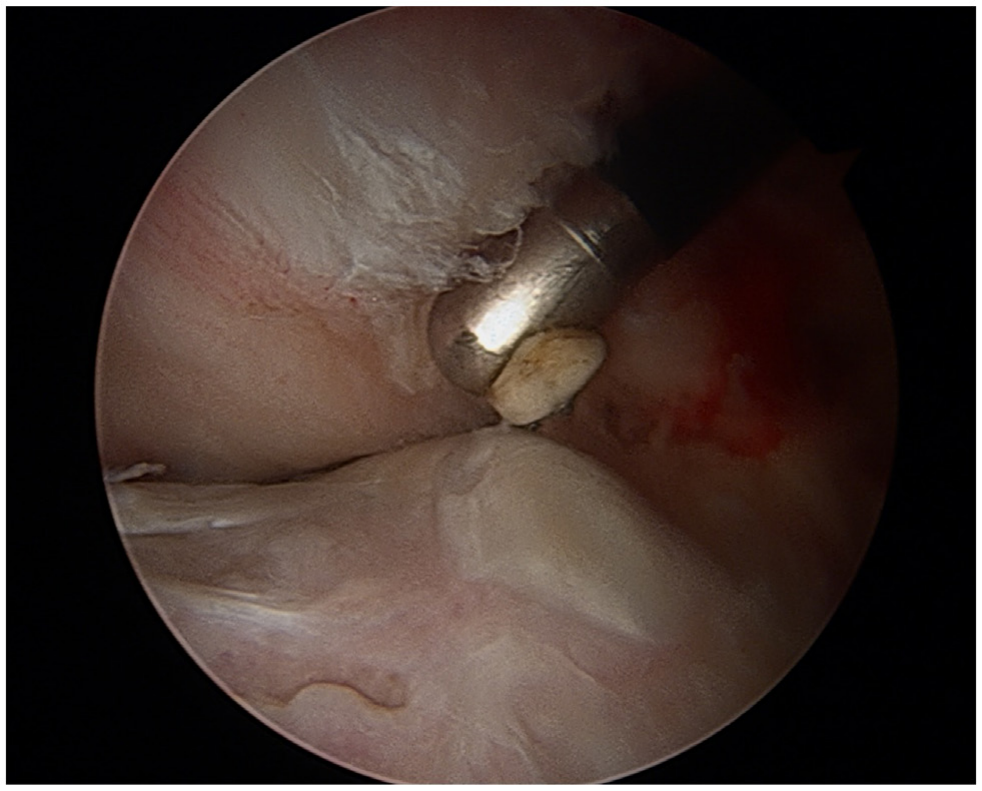
**Arthroscopic view of a cam lesion, right hip**.

**Figure 5:**
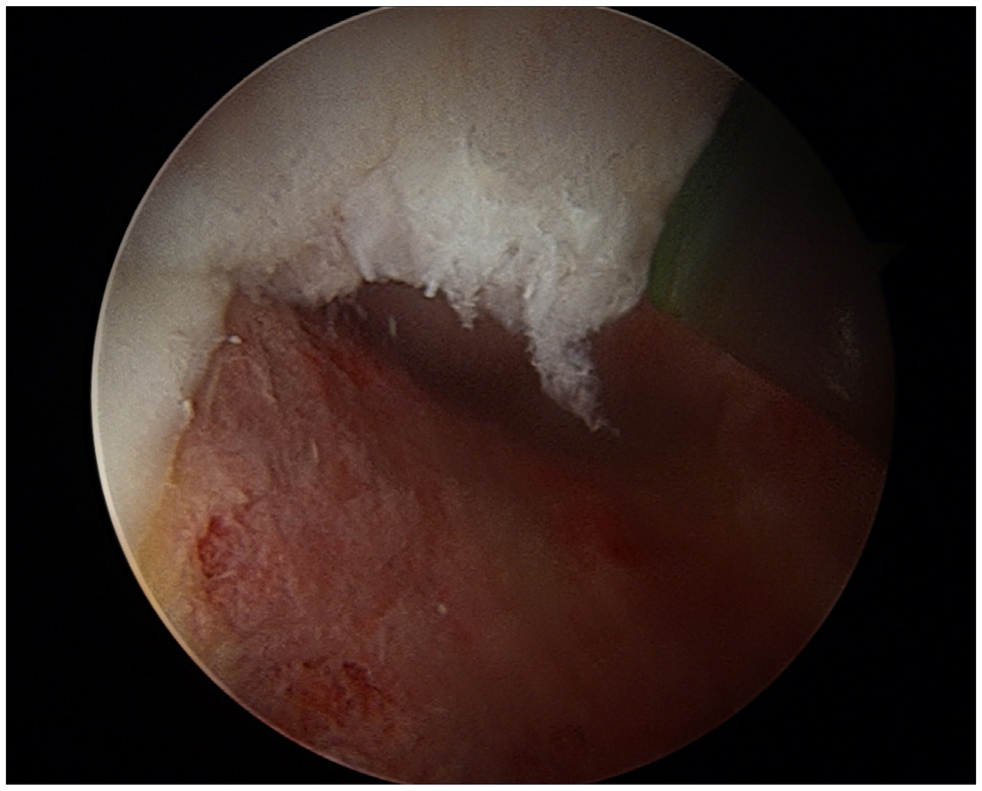
**Arthroscopic view of the hip following cam resection**.

In patients with FAI, treatment of the impingement lesion may restore the femoral head–neck junction offset to a more physiologic state, and relieve stress on nearby muscle groups (4). Surgical techniques include open femoroacetabular osteoplasty, hip arthroscopy, or combined arthroscopy with limited open osteoplasty. FAI is confirmed visually, labral and chondral injuries are identified, debrided, or repaired. Normal bony anatomy is restored through resection of the cam lesion or with acetabular rim trimming (Figures 4 and 5). A burr is used to resect the pincer deformity, and if extra-articular impingement is present, the distal portion of the AIIS is resected. The cam lesion is then addressed with a burr. As the cam lesion can also be focal or more global anteriorly, a three-dimensional computed tomography scan is used to help assess the margins and the lesion. Intraoperatively, the use of fluoroscopy is helpful to confirm that the resection is complete. The ultimate goal is to preserve the hip joint and increase range of motion to 120° of flexion and 40° of rotation (7).

There is some evidence that surgical therapy for both CMI/AP and FAI has shown optimal results than either alone. A case series by Larson et al. demonstrated improved postoperative outcome scores and unrestricted return to sports in 89% of patients who underwent concurrent or eventual surgical treatment of both CMI/AP and FAI (17). However, to the best of our knowledge, there is no literature comparing simultaneous and staged surgical therapy. The authors of this paper previously treated all patients with both hip arthroscopy and laparoscopic inguinal hernia repair simultaneously; however, they now decide which entity is more symptomatic and address that first based on history, physical examination, and advanced imaging. If symptoms persist after the more symptomatic lesion is addressed, the authors address the other lesion.

A multidisciplinary approach between family and sports medicine, and orthopedic surgery and general surgery is essential for improved outcomes in CMI/AP. As FAI and CMI/AP are often linked, awareness of CMI/AP symptoms by orthopedists and FAI symptoms by general surgeons is critical, and the initial evaluator may be one of these many specialists.

## Conclusion

In conclusion, chronic groin pain is often difficult to diagnose and treat in the young athletic population. FAI may lead to compensatory stresses in the biomechanics of the groin. Excessive strains may lead to secondary injury to the posterior inguinal wall, resulting in symptomatic CMI/AP. It is reasonable to consider both overlapping disease processes before surgical intervention is performed.

## Author Contributions

All authors have contributed in writing and editing the manuscript.

## Conflict of Interest Statement

The authors declare that the research was conducted in the absence of any commercial or financial relationships that could be construed as a potential conflict of interest.
